# Computational and pharmacological investigation of novel 1,5-diaryl-1,4-pentadien-3-one derivatives for analgesic, anti-inflammatory and anticancer potential

**DOI:** 10.22038/ijbms.2018.31261.7536

**Published:** 2019-01

**Authors:** Muhammad Sheraz Tariq, Arif-ullah Khan, Amber Mahmood Minhas, Edson Rodrigues Filho, Zia ud Din, Aslam Khan

**Affiliations:** 1 Riphah Institute of Pharmaceutical Sciences, Riphah International University, Islamabad, Pakistan; 2 LaBioMMi, Department of Chemistry, Federal University of São Carlos, CP 676, 13.565-905, São Carlos, SP, Brazil; 3 Department of Chemistry, Woman University Swabi, Guloo Dehri, Topi Road, 23340 Swabi, KPK, Pakistan; 4 Basic Sciences Department, College of Science and Health Professions-(COSHP-J) King Saud bin Abdulaziz University for Health Sciences, Jeddah, Kingdom of Saudi Arabia

**Keywords:** Analgesic, Anticancer, Anti-inflammatory, *In silico* studies, Mice

## Abstract

**Objective(s):**

The novel 1,5-diaryl-1,4-pentadien-3-one derivatives were studied for analgesic, anti-inflammatory and anticancer potential to establish their role in pain, inflammatory disorders and cancer.

**Materials and Methods:**

Two 1,5- diaryl-1,4-pentadien-3-one derivatives: (1E,4E)- 5-(4-fluoro phenyl)-1-(4-methoxyphenyl)- 2-methylpenta-1,4-dien-3-one (A2K2A17) and (1E,4E)-5-(4-nitrophenyl)-1-(4-nitrophenyl)-2-ethylhexa-1,4-dien-3-one (A11K3A11) were synthesized and characterized via 1H NMR and 13C NMR techniques. Molecular docking, anti-inflammatory, analgesic and anticancer activities were performed using Auto Doc Vina, carrageenan mediated paw edema and formalin induced chronic inflammation, acetic acid induced writhings and hotplate assay and brine-shrimp lethality assay.

**Results:**

A2K2A17 and A11K3A11 showed high computational affinities (binding energy > -9.0 Kcal/mol) against COX-1, kappa receptor and braf kinase domain. A2K2A17 and A11K3A11 exhibited moderate docking affinities (binding energy > -8.0 Kcal/mol) against COX-2, human capsaicin receptor, tumor necrosis factor, lipoxygenase, colony stimulating factor, delta receptor, cyclin dependent protein kinase-2, mitogen activated kinase, mu receptor and kit kinase domain. A2K2A17 and A11K3A11 possess low docking affinities (binding energy > -7.0 Kcal/mol) against purinoceptor, platelets-derived growth Factor-1 and vascular-endothelial growth factor. In analgesic activity, A2K2A17 (1-30 mg/kg) and A11K3A11 (1-10 mg/kg) decreased acetic acid induced writhes and prolonged the latency time (*P*<0.01, *P*<0.001 vs saline group) respectively. A2K2A17 (10-30 mg/kg) and A11K3A11 (1-10 mg/kg) reduced carrageenan as well as formalin mediated edema (*P*<0.01, *P*<0.001). A2K2A17 found effective for cytotoxicity assay with LC_50_ value 1.5 µg/ml.

**Conclusion:**

The *in silico*, *in vitro *and *in vivo* studies on A2K2A17 and A11K3A11 reports their computational binding affinities against targets as well as the analgesic, anti-inflammatory and the anticancer effects.

## Introduction

Pain is an unpleasant sensation which is associated with tissue damage (1). Noxious effects such as ulceration, gastrointestinal bleeding by nonsteroidal anti-inflammatory drugs and drowsiness, nausea and tolerance by opioid analgesic limits their use in pain management (2). 

Inflammation is the reaction of living tissues to damage. It includes different events such as activation of enzyme, release of inflammatory mediator and fluid extravasation, migration of cell, tissue breakdown and repair (3, 4). Inflammatory ailments remains one of the major health concerns (5, 6). The adverse effects with nonsteroidal anti-inflammatory drugs (NSAID’s) such as gastric lesions, dependence and tolerance produced by opiates, use of NSAID’s and opiates has not been effective in all cases (7, 8). 

Cancer is diverse group of progressive disorders, characterized by the abnormal and rapid proliferation and is a major problem worldwide. To cope with this problem new site selective drug discovery and development is required (9). 

**Figure 1:**
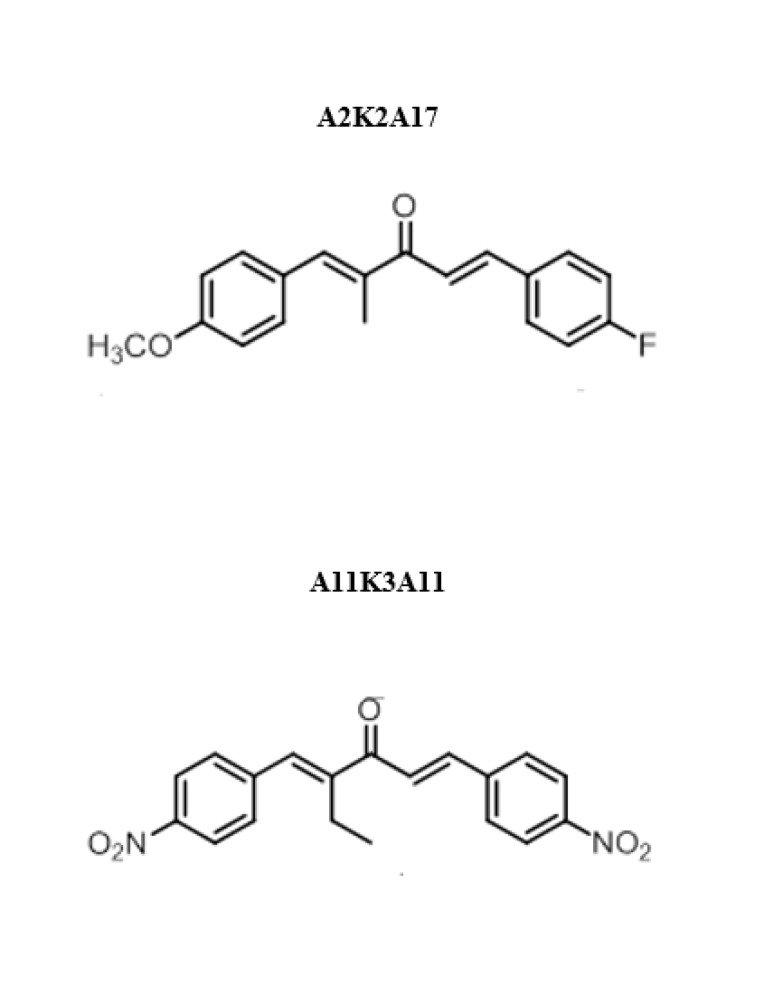
Chemical structure of (1E,4E)-5-(4-fluorophenyl)-1-(4-methoxyphenyl)-2-methylpenta-1,4-dien-3-one (A2K2A17) and (1E,4E)-5-(4-nitrophenyl)-1-(4-nitrophenyl)-2-ethylhexa-1,4-dien-3-one (A11K3A11)

The 1,5 -diarylpentanoid dibenzylidene acetone is the parent structure having an acyclic di-enone attached with aryl groups at b-position. These structures are similar to those of the curcuminoid (1, 7-diaryl heptanes) and the chalcone (1, 3-diaryl propanes). The synthetic chalcone have shown different pharmacological activities; antitumor (10), antioxidant (11) and anti-inflammatory (12, 13). Two 1, 5- diaryl- 1,4-pentadien-3-one derivatives are: (1E,4E)-5-(4-fluorophenyl)-1-(4-methoxyphenyl)-2-methylpenta-1,4-dien-3-one (A2K2A17) and (1E, 4E)-5-(4-nitro phenyl)-1-(4-nitrophenyl)-2-ethylhexa-1,4-dien-3-one (A11K3A11) were synthesized. A2K2A17 and A11K3A11 were studied for analgesic, anti-inflammatory and anticancer effects, using different computational and pharmacological assays. The structures of A2K2A17 and A11K3A11 are shown in Figure 1. 

## Materials and Methods

***Chemicals***

Acetic acid (DAEJUNG Reagents Chemicals), carrageenan (Sigma-Chemicals Co, St-Louis, USA), dimethyl sulphoxide (DMSO), diclofenac (Olive Labs National industrial zone, Islamabad, Pakistan), Formalin (BDH Laboratory supplies, Poole, England), tramadol (Searle company limited F-319, Karachi, Pakistan), methotrexate and ethanol. 

***Test animals***

Mice 25-30 g (Balb-C, n=5 in each group) were kept according to standard protocols (25 ± 2˚C), with natural duration of Light/Dark cycle, each of 12 hrs. Healthy diet was given to mice and water* ad libitum*. The whole study was performed according to the protocols of Animal Resources Institute, Life Science University, National Research Council (NRC 1996), with prior approval by Ethics Committee of RIPS (Riphah Institute of Pharmaceutical Sciences) with Reference no; REC/ RIPS-2016/0012.

***Synthesis***

**Scheme 1:**
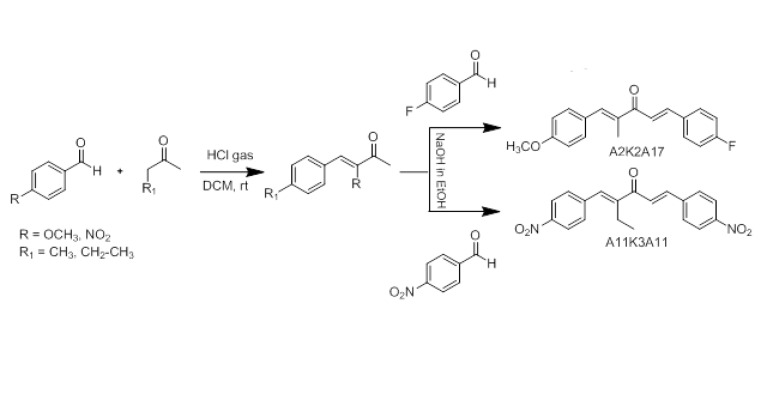
Synthesis of (1E,4E)-5-(4-fluorophenyl)-1-(4-methoxyphenyl)-2-methylpenta-1,4-dien-3-one (A2K2A17) and (1E,4E)-5-(4-nitrophenyl)-1-(4-nitrophenyl)-2-ethylhexa-1,4-dien-3-one (A11K3A11)

The reaction of *p*-methoxy benzaldehyde with 2-butanone in the presence of HCl gas in dichloromethane produces intermediate compounds. The intermediate is further reacted with *p*-Flouro benzaldehyde in ethanol yielding compound A2K2A17. The synthesis of A2K2A17 is reported (14). A11K3A11 is synthesized by first reacting *p*-nitro benzaldehyde with 2-pentanone producing intermediate A11K3, which on further reaction with *p*-nitrobenzaldehyde produces compound A11K3A11 as shown in Scheme 1. Chemical characterization of A11K3A11 was carried out based on the analysis of spectroscopic and crystallographic data. 

***Computational analysis***

Docking is a tool for computational analysis, which is used to investigate affinity and interaction between target protein and ligand (15). We used Auto Dock Vina program for docking study through PyRx. Affinity was determined using interactions of ligand with receptor complex and expressed in the form of binding-energy (E value, Kcal/mol). The 3D-structures of A2K2A17 and A11K3A11 were prepared through Bioviadiscovery Studio Visualizer (DSV) and saved as PDB format. The 3D-structures of target proteins were taken from http://www.rcsb.org/pdb../home.do. The proteins target involved in pain, inflammation and cancer pathways are cyclo-oxygenase-1 (COX-1, PDB-ID: 3N8X), cyclo-oxygenase-2 (COX-2, PDB-ID: IPXX), mu receptor (PDB-ID: 5CIM), kappa receptor (PDB-ID: 4DJH), delta receptor (PDB-ID: 4EJ4), human capsaicin receptor (HCR, PDB-ID: 3J9J) and purinoceptor-3 (P2X3, PDB-ID: 5SVL), C-4 synthetase (PDB-ID: 2UUH), tumor necrosis factor (TNF, PDB-ID: 1TNF), lipooxygenase (5-LOX, PDB-ID: 3O8Y), colony stimulating factor (CSF, PDB-ID: 3UF2), cyclin dependent protein kinase-2 (CDPK-2, PDB-ID: 1HCL), mitogen activated kinase (MAK-ERK-1, PDB-ID: 2ZOQ), insulin like growth factor-1 (ILGF-1, PDB-ID: 1B9G), platelets derived growth factor-1 (PDGF-1, PDB-ID: 1PDG), braf kinase domain (PDB-ID, 4R5Y), vascular endothelial growth factor (VEGF, PDB-ID: 1VPF), nuclear factor kappa (NFK, PDB-ID: 1NFK) and kit kinase domain (PDB-ID: 3G0E). All target proteins were purified by Biovia Discovery Studio Client 2016. The 3D-structures of standard drug molecules were downloaded from the data base (https:/pubchem.ncbi. nlm. nih..gov/search/). Standard analgesic, anti-inflammatory and anti-cancer drugs are aspirin (Pubchem-CID: 2244), apsazepine (Pubchem-CID: 2733484), morphine (Pubchem-CID: 5288826), thymoquinone (Pubchem-CID: 10281), vemurafenib (PubChem-CID: 42611257), sunitinib (Pubchem-CID: 5329102), curcumin (PubCem-CID: 969516) and itraconazole (PubChem-CID: 5793). All these structures were taken in form of .xml and converted to PDB-Format using Open-Babel JUI software. PDB form of both ligand and standard as well as target proteins are converted to PDBQT via Auto Dock Tools (Version1.5.6 Sep_17_14). Both test compounds along with protein targets were loaded in software named as PyRx and then docked against respective targets binding affinity was calculated shown in Kcal/mol. For post docking interaction Discovery studio visualizer was used for number of hydrogen bond (classical/non-classical) and binding residues of amino-acid: Alanine (ALA), Asparagine (ASN), Arginine (ARG), Cysteine (CYS), Aspartic acid (ASP), Glutamine (GLN), Serine (SER), Proline (PRO), Glutamic acid (GLU), Glycine (GLY), Histidine (HIS), Tryptophan (TRP), Threonine (THR), Tyrosine (TYR), Valine (VAL), Leucine (LEU), Lysine (LYS) and Phenylalanine (PHE). 

***Analgesic activity***

*Acetic acid induced writhings*

The analgesic potential of test compounds was determined by acetic acid induced writhings in mice (16). The animals after 12 hr fasting were divided into 5 different groups (5 mice in each group). After 30 min of the administration of A2K2A17 (1, 10, 20 and 30 mg/kg, IP) and A11K3A11 (1, 5 and 10 mg/kg), writhings were induced by intraperitoneal injection (IP) of acetic acid (0.1 ml, 0.7% v/v) to induce pain. Pain perception was recorded in the form of stretch of hind limb and abdominal constriction called as writhe. Some mice showed half writhe. Two half writhes were considered as equal to one full writhe. The writhings were recorded for 20 min. Normal saline (10 ml/kg) was given to saline treated group-negative control while diclofenac (20 mg/kg) was administered to positive control group.

**Figure 2:**
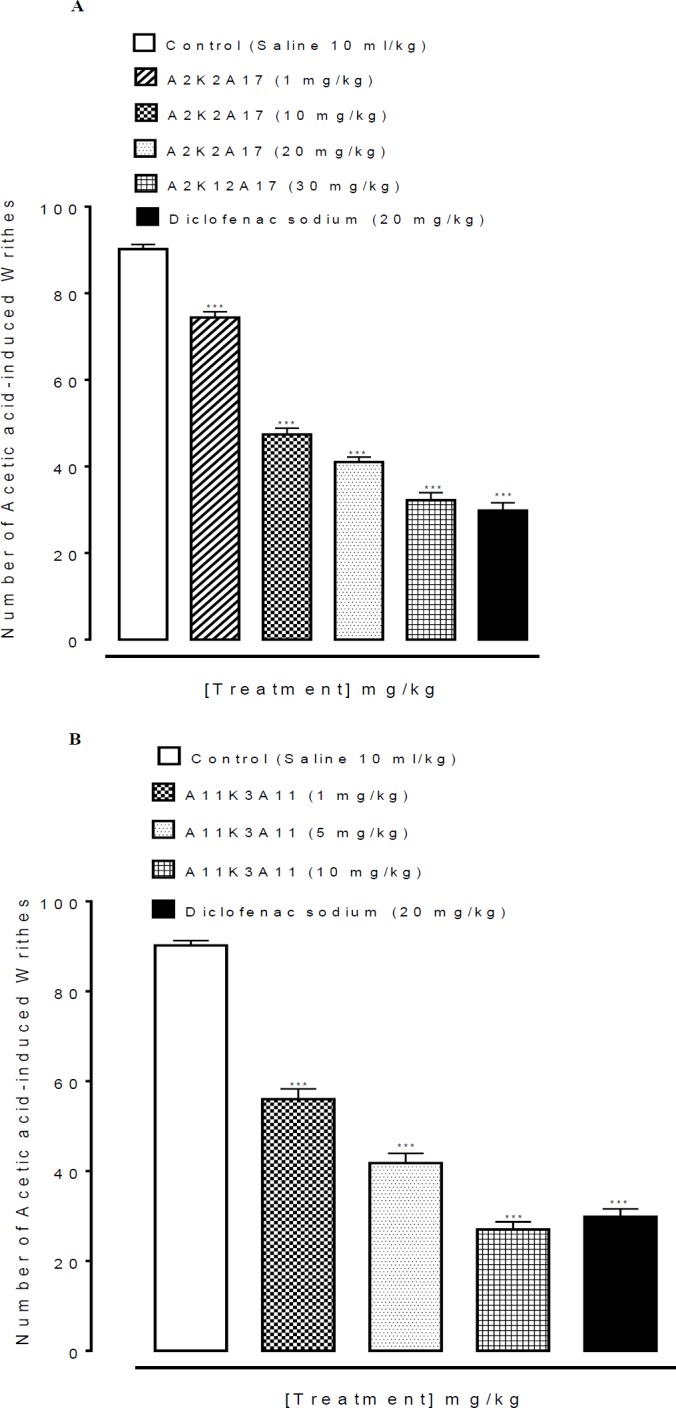
A and B represents the effect of (1E, 4E)-5-(4-fluorophenyl)-1-(4-methoxyphenyl)-2-methylpenta-1,4-dien-3-one (A2K2A17) and (1E,4E)-5-(4-nitrophenyl)-1-(4-nitrophenyl)-2-ethylhexa-1,4-dien-3-one (A11K3A11) respectively, on acetic acid-induced writhes in mice. Data expressed as mean ± SEM , n=5. ***P˂0.001 vs. saline group, one way analysis of variance with* post hoc* Tukey’s test

**Figure 3:**
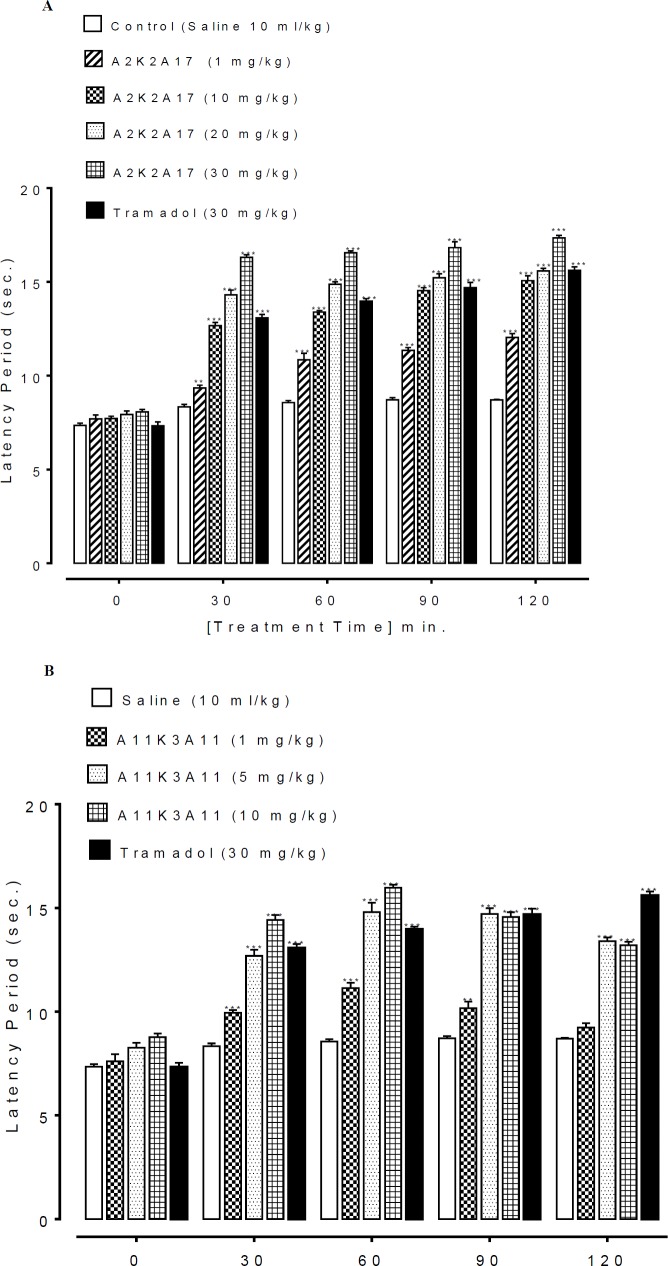
**.** A and B represents the effect of (1E,4E)-5-(4-fluorophenyl)-1-(4-methoxyphenyl)-2-methylpenta-1,4-dien-3-one (A2K2A17) and (1E,4E)-5-(4-nitrophenyl)-1-(4-nitrophenyl)-2-ethylhexa-1,4-dien-3-one (A11K3A11) respectively on latency time in hotplate assay. Data expressed as mean ± SEM, n=5. **P˂0.01, ***P˂0.001 vs. saline group, one way analysis of variance with *post hoc* Tukey’s test

**Figure 4:**
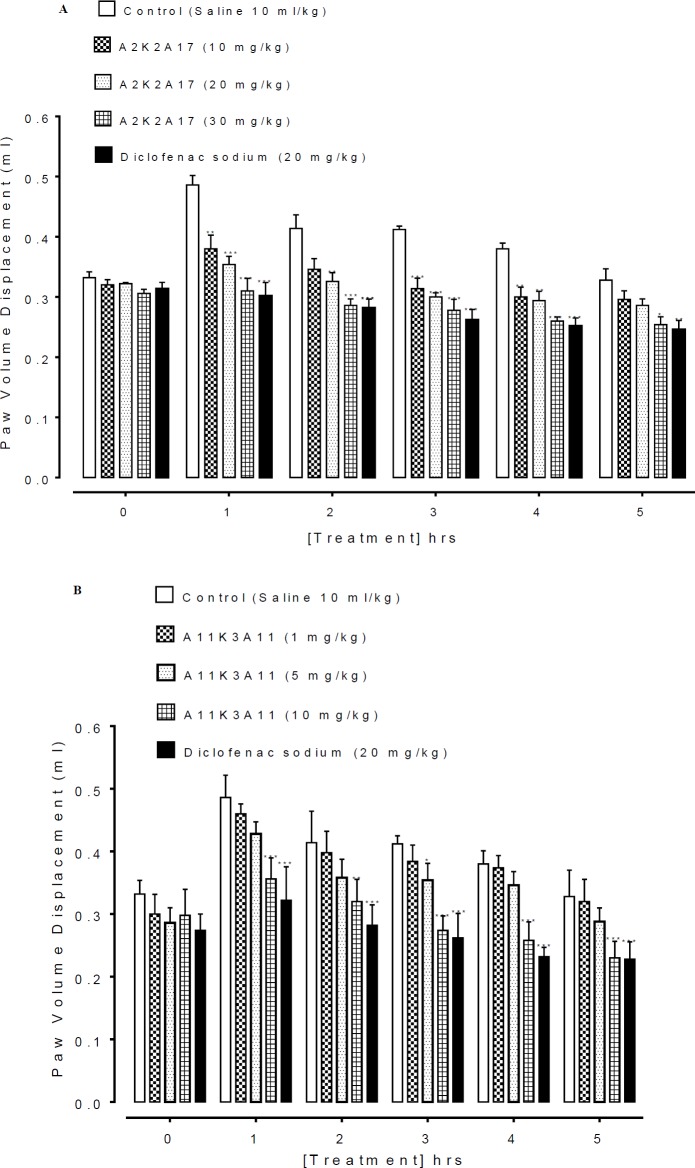
A and B represents the effect of (1E,4E)-5-(4-fluorophenyl)-1-(4-methoxyphenyl)-2-methylpenta-1,4-dien-3-one (A2K2A17) and (1E,4E)-5-(4-nitrophenyl)-1-(4-nitrophenyl)-2-ethylhexa-1,4-dien-3-one (A11K3A11) respectively on carrageenan induced paw edema in mice. Values shown are mean±SEM, n=5. **P˂0.01, ***P˂0.001 vs. saline group, one way analysis of variance with *post hoc* Tukey’s test

**Figure 5:**
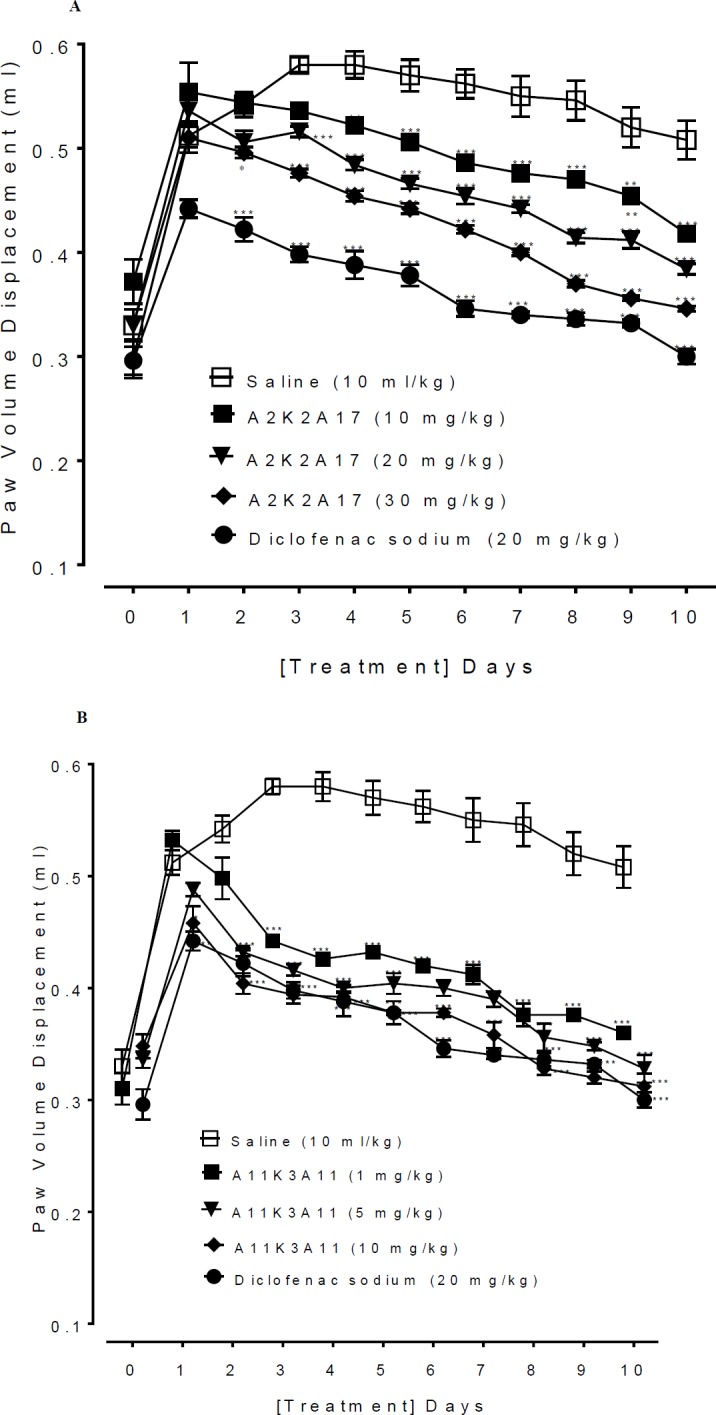
A and B represents the effect of (1E,4E)-5-(4-fluorophenyl)-1-(4-methoxyphenyl)-2-methylpenta-1,4-dien-3-one (A2K2A17) and (1E,4E)-5-(4-nitrophenyl)-1-(4-nitrophenyl)-2-ethylhexa-1,4-dien-3-one (A11K3A11) respectively on formalin induced inflammation in hind paw of mice. Values shown are mean±SEM, n=5. *P˂0.05, **P<0.01, ***P˂0.001 vs. saline group, one way analysis of variance with *post hoc* Tukey’s test

*Hot plate assay*

The mice were distributed into 5 different groups (n=5 in each group). The mice were placed on hot assay plate individually (55±2 ˚C) and observations (licking paws or jumping) at 30, 60, 90 and 120 min were measured. The latency period of test compounds: A2K2A17 (1, 10, 20 and 30 mg/kg) and A11K3A11 (1, 5 and 10 mg/kg), were evaluated via hot plate assay according to the protocols with little modifications (17). Normal saline (10 ml/ kg) was given to control group, tramadol 30 mg/kg (centrally acting opioid analgesic) was used as positive control.

***Anti-inflammatory models***

*Carrageenan mediated paw edema*

The mice were fasted overnight. The displacement of paw was determined using Plethysmometer, Ugo-Basile, before administering any drug (18). The animals were placed into 5 groups (5 mice in each group). Acute inflammation was induced in mice by carrageenan subplantar injection (0.1 ml, 1 % w/v). Saline (10 ml/ kg) was given to negative control group. Half an hr, prior to carrageenan injection, the animals were administered with test compound: A2K2A17 (10, 20 and 30 mg/kg) and A11K3A11 (1, 5 and 10 mg/kg) IP. The standard drug diclofenac sodium (20 mg/kg), was administered to the positive control group. The paw volume was measured at 0 to 5 hr, with 1 hr interval following carrageenan injection (19).

**Table 1:** E-value (Kcal/mol) and post-docking analysis of best pose of (1E,4E)-5-(4-fluorophenyl)-1-(4-methoxyphenyl)-2-methylpenta-1,4-dien-3-one(A2K2A17), (1E,4E)-5-(4-nitrophenyl)-1-(4-nitrophenyl)-2-ethylhexa-1,4-dien-3-one(A11K3A11) and standard drugs with cyclooxygenase-1 (COX-1), cyclooxygenase-2 (COX-2), mu receptor, kappa receptor, delta receptor, human capcaisin receptor (HCR) and purinoceptor-3 (P2X3).

**Target**	**PDB-ID’s**	**A2K2A17**			**A11K3A11**			**Standard drugs**			
		**Kcal/** **mol**	**No of ** **H -Bonds**	**Binding** **Residues**	**Kcal/mol**	**No of** **H-Bond**	**Binding** **Residues**	**Standard**	**Kcal/mol**	**No of** **H -Bonds**	**Binding** ** residues**
**COX-1**	3N8X	-9.1	2	CYS-47 ASP-135	-9.8	7	ARG-49 TRP-323 GLN -461, 44 HIS-43 VAL -48 ARG -469	Aspirin	-6.6	3	GLN-461 PRO-153 ARG-469
**COX-2**	1PXX	-8.5	3	ALA-156 GLN-1543 ARG-44	-8.9	4	ARG-2376 ASN-2537, 3375 PRO- 2538	Aspirin	-7.0	3	TRP-1387 HIS-1388, 1207
**Mu opioid**	5C1M	-8.3	2	HIS-297 LYS-233	-7.9	1	HIS-297	Morphine	-7.2	2	HIS-297 ASP-147
**Kappa opioid**	4DJH	-9.1	2	SER -116 THR-321	-9.7	0	NIL	Morphine	-8.0	0	NIL
**Delta opioid**	1EJ4	-8.4	0	NIL	-8.4	1	LYS-81	Morphine	-7.2	0	NIL
**HCR**	3J9J	-8.5	1	TRP-294	-8.7	1	ARG-177	Capsazepine	-8.2	3	ASN-57 SER-103 TYR-107
**P2X3**	5SVL	-7.2	1	PHE-43	-7.7	2	GLY-40 TYR-49	Capsazepine	-5.4	2	ASP-266 ASN-279

Glutamine (GLN), cysteine (CYS), arginine (ARG), tyrosine (TYR), serine (SER), tryptophan (TRP), alanine (ALA), threonine

(THR), histidine (HIS), asparagine (ASN), valine (VAL), lysine (LYS), proline (PRO), glycine (GLY), phenylalanine (PHE) and aspartic acid (ASP).

**Table 2:** E-value (Kcal/mol) and post-docking analysis of best pose of (1E,4E)-5-(4-fluorophenyl)-1-(4-methoxyphenyl)-2-methylpenta-1,4-dien-3-one (A2K2A17), (1E,4E)-5-(4-nitrophenyl)-1-(4-nitrophenyl)-2-ethylhexa-1,4-dien-3-one(A11K3A11) and standard drugs with C4-Synthetase, tumor necrosis factor (TNF), lipooxygenase (5-LOX) and colony stimulating factor (CSF).

**Target**	**PDB-ID’s**	**A2K2A17**			**A11K3A11**			**Standard drugs**			
		**Kcal/mol**	**No of** **H-Bond**	**Binding residues**	**Kcal/mol**	**No of** **H-Bond**	**Binding residues**	**Standard**	**Kcal/mol**	**No of H-bonds**	**Binding** **residues**
**C4-Synthetase**	2UUH	-7.2	0	NIL	-6.8	0	NIL	Thymoquinone	-3.0	6	SER-57 GLN-19 SER-23
**TNF**	1TNF	-8.1	1	GLU-104	-8.5	2	TYR-115 GLN-102				
**5-LOX**	3O8Y	-8.0	1	ASN-328	-8.4	2	PHE-67 ARG-384	Thymoquinone	-4.0	3	ASN-425 LEU-420 ALA-424
**CSF**	3UF2	-7.7	2	LYS-100 GLU-62	-8.7	1	PHE-67	Vemurafenib	-11.6	3	TYR-6 LEU-85 CYS-93

Glutamine (GLN), cysteine (CYS), arginine (ARG), tyrosine (TYR), serine (SER), alanine (ALA), asparagine (ASN), lysine

(LYS), leucine (LEU), phenylalanine (PHE) and aspartic acid (ASP).

**Table 3:** E-value (Kcal/mol) and post-docking analysis of best pose of (1E,4E)-5-(4-fluorophenyl)-1-(4-methoxyphenyl)-2-methylpenta-1,4-dien-3-one(A2K2A17), (1E,4E)-5-(4-nitrophenyl)-1-(4-nitrophenyl)-2-ethylhexa-1,4-dien-3-one (A11K3A11) and standard drugs with braf kinase domain, cyclin dependent protein kinase-2 (CDPK-2), mitogen activated kinase (MAK-ERK-1), insulin like growth factor-1 (ILGF-1), platelet derived growth factor-1 (PDGF-1), vascular endothelial growth factor (VEGF), nuclear factor kappa b (NFKB) and kit kinase domain

**Target**	**PDB-ID’s**	**A2K2A17**			**A11K3A11**			**Standard Drugs**			
		**Kcal/mol**	**No of** **H-Bonds**	**Binding residues**	**Kcal/mol**	**No of** **H-Bonds**	**Binding residues**	**Standard**	**Kcal/mol**	**No of ** **H-bonds**	**Binding residues**
**Braf kinase domain**	4R5Y	-8.1	1	ASP 594	-9.3	3	THR-529, SER-536, 535	Vemurafenib	-13.7	8	CYS-532 GLN-530 THR-529 GLU-501 ASP-594 HIS-574
**CDPK-2**	1HCL	-8.3	2	LYS-129 GLY-131	-7.8	1	LYS-33	Sunitinib	-7.1	3	ARG-36 THR-41,47
**MAK-ERK-1**	2ZOQ	-8.6	3	ARG-87, 165, 189	-8.4	3	ARG-87, 165, 189	Sunitinib	-7.4	5	ASN-316 THR-223 SER-225, 219 ASP-196
**ILGF-1**	1B9G	-5.8	1	THR-4	-6.0	2	LYS-55 THR-4				
**PDGF-1**	1PDG	-6.3	2	VAL-39 SER-50	-7.1	2	VAL-39 ARG-56	Sunitinib	-6.9	2	VAL-22 SER-50
**VEGF**	1VPF	-7.0	3	CYS-68 GLY-59 LYS-48	-7.8	1	LYS- 48	Itraconazole	-8.0	2	ILE-43 CYS-68
**NFKB**	1NFK	-6.6	3	SER-72 ASN-136 SER-63	-6.8	3	SER-110 LYS-146 THR-143	Curcumin	-7.1	2	ARG-154 THR-143
**Kit kinase domain**	3G0E	-8.8	2	THR-670 ASP-810	-8.5	3	ARG-815 CYS-809 GLY-676	Sunitinib	-8.4	4	GLY-556,812 ARG-815

Glutamine (GLN), cysteine (CYS), arginine (ARG), tyrosine (TYR), serine (SER), glutamic acid (GLU), threonine (THR), histidine (HIS), asparagine (ASN), valine (VAL), lysine (LYS), isoleucine (ILE), glycine (GLY) and aspartic acid (ASP)

**Table 4:** Concentration-dependent (ug/ml) cytotoxic effect of (1E,4E)-5-(4-fluorophenyl)-1-(4-methoxyphenyl)-2-methylpenta-1,4-dien-3-one (A2K2A17), (1E,4E)-5-(4-nitrophenyl)-1-(4-nitrophenyl)-2-ethylhexa-1,4-dien-3-one (A11K3A11) and methotrexate against brine shrimps

**Test samples**	**Concentration (ug/ml)**							**LC**_50 _ **values** **(ug/ml)**
	**1 **	**3 **	**5 **	**10 **	**100 **	**300 **	**1000 **	
A2K2A17	33.3 ± 0.33	73.3 ± 0.33	90.0 ±0.00	96.6 ± 0.33	100 ± 0.00	100 ±0.00	100 ± 0.00	1.50
A11K3A11	20 ± 0.00	20.66 ±0.33	30.33 ±0.33	43.3 ± 0.33	46.6 ±0.33	66.6 ±0.33	76.6 ± 0.33	107.29
Methotrexate	15.4 ± 5.1	48.3 ± 4.5	59.7± 3.5	81.3 ± 3.50	91.7 ± 6.7	100 ± 0.0	100 ± 0.0	3.39

Values shown as % mean ± SEM, n=5.

*Formalin induced edema*

The anti-inflammatory potential against chronic inflammation was determined using formaldehyde mediated edema (20). The animals were divided into 5 groups (n=5). The base line paw volume displacement was determined using Plethysmometer. Inflammation induced by sub-aponeurotic administration of formalin (0.1 ml, 2% v/v formaldehyde) in the left hind paw on first day and third day. Normal saline (10 ml/kg) was given to negative control group. The paw volume displacement of A2K2A17 (10, 20 and 30 mg/kg) and A11K3A11 (1, 5 and 10 mg/kg) administered IP, were measured up to 10 days. Diclofenac sodium (20 mg/kg) was used as the positive control.

***Cytotoxicity assay***

*Brine-shrimp lethality test*

The cytotoxicity of test compound was measured using brine shrimp cytotoxicity assay (21). This method requires the preparation of simulated sea water for the hatching of brine shrimp eggs. The newly hatched larvae (nauplii) were observed in the lighter compartment because of their photosensitive nature. Living nauplii (n=10) were collected by Pasteur’s pipette and added to the vials of 96 well plate then simulated sea water (5 ml) was added to each vial. These nauplii were subjected to serial dilution of the each of the test compound (1-1000 ug/ml) in 5% DMSO and the number of larvae killed were counted after 24 hr of incubation using 3×magnifying glass. The lethal concentrations (LC_50_) were then calculated for all of test compounds. 

***Statistical evaluation***

Data expressed as the mean±SEM (Standard Error of Mean). The results were evaluated by GraphPadprism (Graphpad, San-Diego/CA, USA) with one-way analysis of variance (ANOVA), followed by Tukey’s *post hoc* test. * P*<0.05 was considered different significantly.

## Results

***Spectral analysis of A11K3A11***

^1^H NMR (400 MHz, CDCl_3_) δ 8.32 – 8.24 (m, 4H), 7.75 (t, *J* = 12.1 Hz, 3H), 7.57 (d, *J* = 8.6 Hz, 2 H), 7.52 – 7.41 (m, 2H), 2.65 (q, *J* = 7.5 Hz, 2 H), 1.16 (t, *J* = 7.5 Hz, 3H). The ^13^C NMR (101 MHz, CDCl_3_) δ 191.46 (1C), 148.72 (1C), 147.59 (1C), 142.22 (1C), 141.40 (1C), 141.07 (1C), 135.87 (1C), 129.98 (2C), 129.03 (3C), 125.85 (1C), 124.38 (2C), 124.03 (2C), 20.77 (1C), 13.65 (1C).

***Docking evaluation***

Table 1 summarized the E-value (Kcal/mol), hydrogen bond and binding residues of A2K2A17 and A11K3A11 with target proteins (COX-1, COX-2, mu receptor, kappa receptor, delta receptor, HCR and P2X3) involved in pain pathways along with standard drugs. The E-value (Kcal/mol), hydrogen bonds and binding residues of A2K2A17 and A11K3A11 with target proteins: C-4 synthetase, TNF, 5-LOX and CSF involved in inflammation along with standard drugs are shown in Table 2. The E-value, hydrogen bonds and binding residues of A2K2A17 and A11K3A11 with target proteins: braf kinase domain, CDPK-2, MAK-ERK-1, ILGF-1, PDGF-1, VEGF, NFKB and kit kinase domain involved in cancer pathway along with standard drugs are shown in Table 3. 

***Effect on acetic acid-induced writhings***

Saline (10 ml/kg) treated group showed 90.20 ± 1.068 numbers of writhes. The writhes count of A2K2A17 (1, 10, 20 and 30 mg/kg) treated groups decreased to 74.40 ± 1.32, 47.40 ± 1.43, 41.00 ± 1.140 and 32.200 ± 1.77 (*P*<0.001 vs saline group) (Figure 2A). The writhes count of A11K3A11 (1, 5 and 10 mg/kg) decreased to 56 ± 2.280, 41.80 ± 2.154, and 27 ± 1.70 (*P*<0.001 vs. saline group) respectively (B). Diclofenac sodium (20 mg/kg) decreased numbers of writhes to 28.80 ± 1.77 (*P*<0.001 vs. saline group).

***Effect on latency time in hot plate assay***

The latency time of saline (10 ml/kg) treated group at 0, 30, 60, 90, 120 min was 7.35 ± 0.12, 8.33 ± 0.13, 8.56 ± 0.10, 8.71 ± 0.10 and 8.70 ± 0.03 sec respectively. A2K2A17 dose dependently (1, 10, 20 and 30 mg/kg) prolonged latency time (*P*<0.01 vs. saline group) against thermal pain generation (Figure 3A). A11K3A11 dose dependently (1, 5 and 10 mg/kg) prolonged latency time (*P*<0.01 vs. saline group) against thermal pain generation (Figure 3B). Tramadol (30 mg/kg) reduced the latency (*P*<0.001 vs. saline group). 

*Effect of A2K2A17 and A11K3A11 on Carrageenan mediated paw edema*

A2K2A17 (10-30 mg/ kg) reduced the carrageenan mediated inflammation in a dose dependent way (Figure 4A). The subplantar injection of carrageenan produce edema which progressively increases with time in the saline treated control group. Treatment of animal with A2K2A17 (10- 30 mg/kg) and A11K3A11 (1-10 mg/kg) decreased carrageenan mediated paw inflammation (*P*<0.05, *P*<0.01, *P*<0.001 vs saline group) as presented in Figure 4 (A and B) respectively. Similarly, diclofenac (20 mg/kg) decreased the carrageenan induced inflammation in paw.

*Effect on formalin mediated inflammation*

A significant increase in the left hind paw thickness was observed in the saline treated control group after formalin injection. Continuous treatment with A2K2A17 (10-30 mg/kg) and A11K3A11 (1-10 mg/kg) remarkably reduces paw edema (*P*<0.05, *P*<0.01, *P*<0.001 vs saline). The reduction in paw thickness was observed from the day 1^st^ and throughout the time period of study (10 days), compared with saline treated group shown in Figure 5 (A and B) respectively. Diclofenac at dose of 20 mg/kg reduces the paw edema (*P*<0.001 vs saline group).

***Effect on brine shrimp lethality***

A2K2A17, A11K3A11 and methotrexate exhibited concentration-dependent (1, 3, 5, 10, 100, 300 and 1000 ug/ml) cytotoxic effect against brine shrimps (Table 4). The larvae killed by A2K2A17 and A11K3A11, with LC_50_ value of 1.50 µg/ml and 107.29 µg/ml respectively. The cytotoxic effect by methotrexate occurs at LC_50_ value of 3.39 ug/ml.

## Discussion

Molecular docking has gained valuable importance in the field of drug development. The main purpose of docking is to get the preliminary information about affinity of any compound before the start of *in vivo* experiment. The docking of novel 1, 5-diaryl-1, 4-pentadien-3-one derivative i.e, A2K2A17 and A11K3A11 were carried out using Autodock Vina program through PyRx (22). Docking tool was preliminary tool used to check the affinity of ligands to their respective protein targets. These interactions may be in the form of hydrogen bonds, hydrophobic interaction and Van der Waal forces (23). Hydrogen bonding has significant role in the formation of ligand protein complex. In this study we assessed affinity of ligand using three parameters: E-value, number of hydrogen bond and amino acid residue against the protein targets involve in pain, inflammation and cancer. A2K2A17 order of affinity against target protein was found as: COX-1 > kappa receptor > braf kinase domain > COX-2 > HCR = CSF > kit kinase domain > TNF > MAK-ERK-1 > lipooxygenase > delta receptor > mu receptor > CDPK > VEGF > P2X3 > PDGF-1 > NFK > C-4 synthetase > ILGF-1. A11K3A11 order of affinity against target proteins was found as: COX-1 > kappa receptor > kit kinase domain > MAK-ERK-1 > COX-2 > HCR > delta > mu receptor > CDPK > braf kinase domain = TNF > lipooxygenase > CSF > P2X3 > C4 synthetase > VEGF > NFK > PDGF-1`> ILGF-1. 

The analgesic activity was tested with two protocols; Acetic acid induce writhes method and hot plate assay, to evaluate the peripheral as well as central effects of analgesia (24). A2K2A17 and A11K3A11 showed dose-dependent analgesic effect. A2K2A17 is more effective in increasing the latency period compared to A11K3A11, while both compounds were equally effective against acetic acid induce writhing. Acetic acid produces nociception by release of chemical mediator such as histamine, serotonin, bradykinins, prostaglandin and substance-P due to induction of COX-2 that results in increased pain sensitivity after acetic acid injection (25). Generally used nonsteroidal anti-inflammatory drug (NSAID’s) such as diclofenac sodium and indomethacin have shown anti-nociceptive effect, by decreasing the production of prostaglandin through blockage of COX-2.

Carrageenan induced inflammation is a well-known experimental model to determine the anti-inflammatory activity (26). Carrageenan produces inflammation via release of several mediator of inflammation (such as histamine, serotonin, prostaglandins and bradykinin) in early and late phases (27). Several studies show that substances which decreases the carrageenan-induced edema, produces the prostaglandins synthesis inhibition by the cyclo-oxygenase (COX) enzyme inhibition (28). A2K2A17 and A11K3A11 caused carrageenan produced paw edema inhibition in a dose dependent fashion similar to the effect caused by a standard NSAID i,e diclofenac sodium. The NSAID’s reduces pain, swelling and inflammation through inhibition of COX-enzyme in the arachidonic acid pathway (29). A2K2A17 and A11K3A11 were effective in inhibiting carrageenan mediated paw edema. On the basis of these results, it can be said that the anti-inflammatory action of A2K2A17 and A11K3A11 occurs through the inhibition of prostaglandin synthesis. A2K2A17 and A11K3A11 were further investigated against chronic inflammation using formalin induced edema (30). Two injections sub-aponeuroticaly were used to induce the chronic inflammation, the inflammation is characterized by increase in paw-thickness and increase volume, responses of chronic inflammation (20). The test compounds were effective in reduction of the formalin induced cellular damage. The test compounds were investigated for *in vitro* cytotoxicity using brine shrimps assay. The lethality of brine shrimp is because of less developed membrane susceptibility to cytotoxic chemical (15). A2K2A17 was found to be more effective with 1.5 µg/ml LC_50_ value as compared to A11K3A11, with 107.29 µg/ml LC_50_ value. Moreover the anticancer potential needs further investigation by screening the test compounds through cell line assays using human cancer cells.

## Conclusion

Computational studies reveal binding affinities of 1,5-diaryl-1,4-pentadien-3-one derivatives: A2K2A17 and A11K3A11 against proteins targets involved in the pathogenesis of pain, inflammation and cancer as well as exhibit analgesic, anti-inflammatory and anticancer activities which explore their therapeutic effectiveness in pain, inflammatory disorders and tumor. Further studies are warranted to determine safety profile, pharmacokinetics and pharmacodynamics of test compounds to establish them as lead molecules.

## References

[1] Loeser JD, Melzack R (1999). Pain: an overview. The Lancet.

[2] Zulfiker AHM, Rahman MM, Hossain MK, Hamid K, Mazumder MEH, Rana MS (2010). In vivo analgesic activity of ethanolic extracts of two medicinal plants-Scoparia dulcis L and Ficus racemosa Linn. Biol Med.

[3] Vane J, Botting R (1995). New insights into the mode of action of anti-inflammatory drugs. Inflamm Res.

[4] Perianayagam JB, Sharma S, Pillai K (2006). Anti-inflammatory activity of Trichodesma indicum root extract in experimental animals. J Ethnopharmacol.

[5] Yeşilada E, Üstün O, Sezik E, Takaishi Y, Ono Y, Honda G (1997). Inhibitory effects of Turkish folk remedies on inflammatory cytokines: interleukin-1α, interleukin-1β and tumor necrosis factor α. J Ethnopharmacol.

[6] Li RW, Myers SP, Leach DN, Lin GD, Leach G (2003). A cross-cultural study: anti-inflammatory activity of Australian and Chinese plants. J Ethnopharmacol.

[7] Dharmasiri MG, Jayakody JRAC, Galhena G, Liyanage SSP, Ratnasooriya WD (2003). Anti-inflammatory and analgesic activities of mature fresh leaves of Vitex negundo. J Ethnopharmacol.

[8] Park JH, Son KH, Kim SW, Chang HW, Bae K, Kang, SS et al (2004). Anti-inﬂammatory activity of Synurus deltoides. Phytother Res.

[9] Amin KM, Eissa AA, Abou-Seri SM, Awadallah FM, Hassan GS (2013). Synthesis and biological evaluation of novel coumarin-pyrazoline hybrids endowed with phenylsulfonyl moiety as anti-tumor agents. Eur J Med Chem.

[10] Cabrera M, Simoens M, Falchi G, Lavaggi ML, Piro OE, Castellano EE (2007). Synthetic chalcones, flavanones, and flavones as antitumoral agents: Biological evaluation and structure–activity relationships. Bioorganic Med Chem.

[11] Anto RJ, Sukumaran K, Kuttan G, Rao MNA, Subbaraju V, Kuttan R (1995). Anticancer and antioxidant activity of synthetic chalcones and related compounds. Cancer Lett.

[12] Elias D, Beazely M, Kandepu N (1999). Bioactivities of chalcones. Curr Med Chem.

[13] Araico A, Terencio MC, Alcaraz MJ, Dominguez JN, León C, Ferrándiz ML (2007). Evaluation of the anti-inflammatory and analgesic activity of Me-UCH9, a dual cyclooxygenase-2/5-lipoxygenase inhibitor. Life Sci.

[14] Din ZU, Fill TP, de Assis FF, Lazarin-Bidóia D, Kaplum V, Garcia FP et al (2014). Unsymmetrical 1, 5-diaryl-3-oxo-1, 4-pentadienyls and their evaluation as antiparasitic agents. Bioorganic Med Chem.

[15] Meyer BN, Ferrigni NR, Putnam JE, Jacobsen LB, Nichols DE, McLaughlin JL (1982). Brine shrimp: a convenient general bioassay for active plant constituents. Planta Medica.

[16] Ahmed F, Selim MST, Das AK, Choudhuri MSK (2004). Anti-inflammatory and antinociceptive activities of Lippianodiflora Linn. Pharmazie.

[17] Adzu B, Amos S, Kapu S, Gamaniel K (2003). Anti-inflammatory and anti-nociceptive effects of Sphaeranthus senegalensis. J Ethnopharmacol.

[18] Padilha MM, Vilela FC, Rocha CQ, Dias MJ, Soncini R, dosSantos MH (2010). Antiinflammatory properties of Morus nigra leaves. Phytother Res.

[19] Ray SD, Ray S, Zia-Ul-Haq M, DeFeo V, Dewanjee S (2015). Pharmacological basis of the use of the root bark of Zizyphus nummularia Aubrev (Rhamnaceae) as anti-inflammatory agent. BMC Complement Altern Med.

[20] Cho AE, Guallar V, Berne BJ, Friesner R (2005). Importance of accurate charges in molecular docking: quantum mechanical/molecular mechanical (QM/MM) approach. J Comput Chem.

[21] Bibi G, Ullah N, Mannan N, Mirza B (2011). Antitumor, cytotoxic and antioxidant potential of Aster thomsonii extracts. Afr J Pharm Pharmacol.

[22] Dallakyan S, Olson AJ (2015). Small-molecule library screening by docking with PyRx. Chemical Biology.

[23] Ayyappa B, Kanchi S, Singh P, Sabela MI, Dovey M, Bisetty K (2015). Analytical evaluation of steviol glycosides by capillary electrophoresis supported with molecular docking studies. J Iran Chem Soc.

[24] Gené RM, Segura L, Adzet T, Marin E, Iglesias J (1998). Heterotheca inuloides: anti-inflammatory and analgesic effect. J Ethnopharmacol.

[25] Bentley GA, Newton SH, Starr JB (1983). Studies on the antinociceptive action of α-agonist drugs and their interactions with opioid mechanisms. Br J Pharmacol.

[26] Bukhari IA, Khan RA, Gilani AUH, Shah AJ, Hussain J, Ahmad VU (2007). The analgesic, anti-inflammatory and calcium antagonist potential of Tanacetum artemisioides. Arch Pharm Res.

[27] Burch RM, DeHaas C (1990). A bradykinin antagonist inhibits carrageenan edema in rats. Naunyn-Schmiedeberg’s Arch Pharmacol.

[28] Selvam C, Jachak SM (2004). A cyclo-oxygenase (COX) inhibitory bi flavonoids from seeds of Semecarpus anacardium. J Ethnopharmacol.

[29] Grosser T, Smyth E, FitzGerald GA, Goodman LS, Gilman A, Brunton LL (2011). Antiinflammatory, antipyretic and analgesic agents. The Pharmacological Basis of Therapeutics.

[30] Igbe I, Ching FP, Eromon A (2010). Anti-inflammatory activity of aqueous fruit pulp extract of Hunteria umbellata K schum in acute and chronic inflammation. Acta Pol Pharm.

